# Myocardial Infarction and the Fine Balance of Iron∗

**DOI:** 10.1016/j.jacbts.2021.06.004

**Published:** 2021-07-26

**Authors:** Rohan Dharmakumar, Anand R. Nair, Andreas Kumar, Joseph Francis

**Affiliations:** a Biomedical Imaging Research Institute, Department of Biomedical Sciences, Cedars-Sinai Medical Center, Los Angeles, California, USA; b Division of Cardiology, Department of Medicine, University of California at Los Angeles, Los Angeles, California, USA; c Division of Cardiology, Department of Medicine, Northern Ontario School of Medicine, Sudbury, Ontario, Canada; d Department of Comparative Biomedical Sciences, School of Veterinary Medicine, Louisiana State University, Baton Rouge, Louisiana, USA

**Keywords:** acute myocardial infarction, heart, iron

**Figure:**
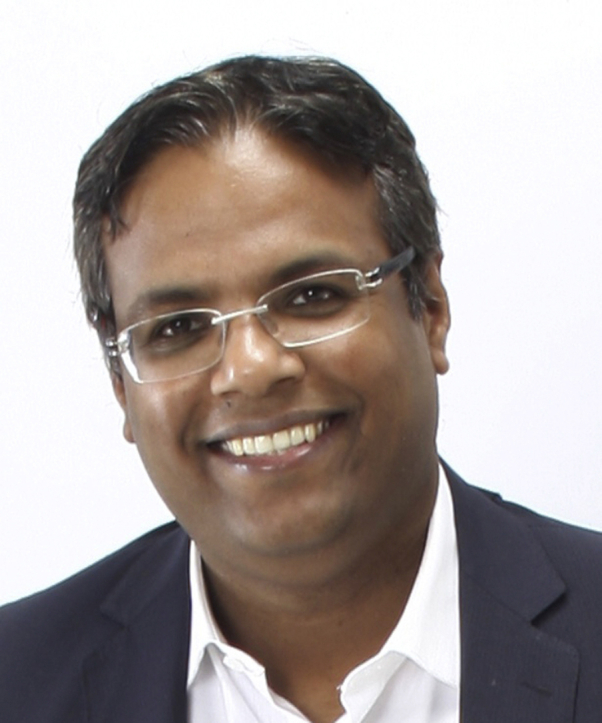

In the heart and in many other organ systems, iron is a recognized double-edged sword as its valence status enables it to catalyze redox reactions that can maintain cellular homeostasis or drive toxic damages. Notably, iron is a central player in cardiac energetics (both in the transport of molecular oxygen and in cellular respiration), but it can also contribute to the death of cardiomyocytes through uncontrolled oxidative stress. On the whole, how iron comes into play in a cellular event and whether it drives a life supporting or compromising process is highly dependent on the physiologic environment.

Although the life-sustaining aspects of iron in the form of molecular transport of oxygen on the backs of hemoglobin complexes and activities of electron transport chain in cellular respiration are well understood, the pathologic aspects of iron are continually being discovered. Importantly, a number of studies have shown that both iron overload and iron deficiency are implicated in heart failure. Cardiac iron overload is commonly observed in a number of pathologies (eg, hemochromatosis) driven by genetic mutations, multiple blood transfusions, or iron supplementation. Although iron is typically complexed with transferrin or ferritin, when there is excessive iron (transferrin saturation >80%), the non–transferrin-bound iron within cardiomyocytes can drive excessive production of reactive oxygen species (ROS) and activation of fibroblasts. These processes can drive myocardial fibrosis, leading to impaired myocardial contractility/relaxation that drives diastolic dysfunction, arrhythmias, ventricular dilation, and systolic dysfunction. Mitigating the systemic cardiac iron overload in the heart with the use of iron chelation therapies have proven to be effective in restoring iron concentrations to normal levels and curbing the progressive decline of cardiac function.

Conversely, iron deficiency can also trigger adverse effects on the heart. Studies have shown that systemic iron deficiency (serum ferritin <100 μg/L) can result from inadequate dietary intake, and functional iron deficiency (100-300 μg/L with transferrin saturation <20%) can result from the inability of the body to utilize circulating iron in an optimal manner. While the relationship between serum iron deficiency and myocardial iron deficiency are not completely understood, nearly 2 out 3 heart failure patients are presented with systemic iron deficiency (1). In line with these clinical observations, one hypothesis that is currently being investigated is whether the diminished cardiac function is driven by systemic iron deficiency compromising cardiac energetics. Recent studies have shown that in these patients, although ferric iron infusions can reduce heart failure hospitalizations (2), iron supplementation in the same patients has failed to improve mortality (1). Thus, the collective findings support the notion that although iron deficiency is common in heart failure, it is a complex problem with much remaining to be understood.

While systemic iron overload or iron deficiency driving myocardial abnormalities in iron concentration has been investigated, the importance of iron in myocardial infarction (MI) has also been recognized. Numerous studies have shown that reperfusion of coronary arteries after appreciable ischemia can raise the concentration of ROS and overwhelm the antioxidant defenses, with the production of ROS being driven by Fenton reactions catalyzed by iron. Extensive ROS activity is known to drive cellular necrosis, apoptosis, and ferroptosis—all contributing to increasing infarct size after ischemia and reperfusion (I/R). In the setting of acute MI, studies that aimed to improve the physiologic environment via iron chelation treatment, mainly involving deferoxamine (a large FDA-approved iron chelator with low cellular permeability), have produced mixed results, with some studies showing improved cardiac function but not reduction in infarct size. Unlike earlier studies investigating total cellular iron, more recent I/R studies in rodents have found that subcellular concentration of iron, particularly mitochondrial iron, can be substantially increased. In support of this hypothesis, one study showed that reducing the mitochondrial iron within the cardiomyocytes with a smaller cell-permeable iron chelator, 2,2′-bipyridyl, is effective in reducing iron and reducing tissue damage in a rodent model (3). Further still, we and others have shown that when I/R culminates in microvascular injury (microvascular obstruction and intramyocardial hemorrhage), that can lead to increased iron concentration within the infarcted myocardium. This increase in iron within MI territories can persist for a long period, driving a proinflammatory milieu that facilitates adverse left ventricular (LV) remodeling (4).

Alternatively, the understanding of how iron overload or deficiency affects acute MI is sparse, but data are emerging. Although iron overload has not been implicated in acute MI, studies suggest that iron deficiency at the time of MI can portend adverse prognosis; however, the molecular pathways that drive such adverse outcomes in iron deficiency have not been elucidated. In this issue of *JACC: Basic to Translational Science*, Inserte et al (5) investigated the effect of iron deficiency in reperfused ST-segment elevation MI patients). To support their observations in patients mechanistically, they used a murine model of I/R. Their studies build on the premise that iron deficiency compromises mitochondrial function, inducible nitric oxide synthase (iNOS) expression and antioxidant capacity. Specifically, they tested the hypothesis that iron deficiency is associated with larger MI size and adverse LV remodeling in reperfused MI patients and investigated whether: 1) iron-deficient mice had altered tolerance to I/R; and 2) whether iron supplementation can rescue the animals from I/R injury. Mechanistically, the authors studied if the susceptibility of the cardiomyocytes to I/R injury under systemic functional iron deficiency was mediated by an attenuated endothelial nitric oxide synthase (eNOS)/soluble guanylyl cyclase (sGC)/protein kinase G (PKG) pathway. Aside from being a well-known cardioprotective pathway, the rationale for studying this specific pathway was based on previous evidence that 1) iron deficiency can induce oxidative/nitrosative stress; and 2) iron is indispensable for the normal functioning of eNOS and sGC enzymes.

The patient study recruited and studied 125 patients with first ST-segment elevation MI, who were mechanically reperfused, of whom 43% had functional iron deficiency. Compared with those without, those with functional iron deficiency had larger MI size (normalized for area at risk based on T2-STIR cardiac magnetic resonance imaging), greater frequency and extent of microvascular obstruction on late gadolinium enhancement and indifferent extent of intramyocardial hemorrhage at 5 days (mean) following reperfusion. Furthermore, experimental data in mice showed that an iron-deficient diet induces oxidative stress and decreases eNOS activity by promoting its proteasomal degradation, thereby resulting in lowered nitric oxide production and attenuated sGC activity. Both supplementation of iron with iron-sucrose and administration of an sGC agonist before I/R independently resulted in a reduced infarct size compared with iron-deficient mice.

This an exciting translational study with novel findings that can have important implications for patients at risk of developing an acute coronary syndrome. The study builds on previous findings and implicates a mechanistic pathway driving the tissue damage in the acute phase. While much of the imaging studies in patients are reasonably well guided, there are some shortcomings. For example, it remains controversial whether T2-STIR (or any T2-based approaches for that matter) can retrospectively estimate the area at risk with sufficient accuracy. Furthermore, the imaging study provides only limited insight into the prevalence of hemorrhagic infarction in the patient population on the basis of T2-STIR images, which is known to have limited sensitivity for detecting hemorrhage.

On a mechanistic level, inhibition of the eNOS/sGC/PKG pathway has been shown to contribute to necrotic cell death. In addition, the same research group has previously reported that the contribution of apoptosis to cell death occurring during myocardial I/R injury is minimal. However, this argument needs to be further supported by experimental data because iron deficiency, by itself, is known to contribute to apoptosis. Therefore, in iron-deficient conditions, involvement of apoptosis and/or other programmed cell death mechanisms cannot be ruled out as yet. Furthermore, in this study, the authors show that cardiomyocytes are affected by iron deficiency. Nonetheless, because eNOS is implicated in mediating the susceptibility to I/R injury, a direct effect of iron deficiency on endothelial cells is worth exploring. Furthermore, although iron supplementation seems to be beneficial in iron-deficient mice after the induction of an I/R injury, supplementation of iron should not be considered as a prophylactic alternative in myocardial ischemia. Recent trials showed clinical benefit of the sGC stimulator vericiguat in a heart failure population, but the effect of sGC stimulators in an I/R setting remains to be explored in larger patient trials. This new drug class opens a treatment avenue affecting the downstream effects of iron deficiency while not targeting iron itself. As already mentioned, iron overload results in severe acute and long-term outcomes for MI patients. Therefore, similar to many other microelements, the amount of iron needs to be within a physiological range for optimal cellular functioning. Thus, iron supplementation as a therapeutic option after MI, even in subjects with iron deficiency, requires diligent monitoring to ensure that its concentration does not become detrimental.

As one of the first translational studies on acute MI in the setting of functional iron deficiency, this study inspires the need for additional insight within the greater context of iron in MI. First, it is unclear how iron deficiency drives microvascular damage and whether iron deficiency, microvascular damage, or a combined effect drives adverse LV remodeling. Studies are also needed for understanding the relationship between acute MIs that take place under conditions of functional iron deficiency and development of post-MI heart failure with iron deficiency; that is, is heart failure with functional iron deficiency as we know it rooted in functional iron deficiency before MI? Moreover, it remains unclear whether cases of acute MI in conditions of functional iron deficit result in compartmental alterations in iron (particularly within the mitochondrion), as under normal conditions of iron; and if so, how do such changes relate to the observations of presented in this study? In spite of these remaining questions, what is starting to emerge is that both iron overload and iron deficiency can drive myocardial damage, unless there is a fine balance.

## Funding Support and Author Disclosures

Dr Dharmakumar is supported by National Institutes of Health/National Heart, Lung, and Blood Institute grants HL133407, HL136578, HL147133, and HL148788. Dr Francis is supported by National Institutes of Health/National Heart, Lung, and Blood Institute grant HL148788. Dr Dharmakumar is an inventor on U.S. patents 14/909,922, 14/125,307, and 15/064,817. All other authors have reported that they have no relationships relevant to the contents of this paper to disclose.

## References

[1] Ponikowski P., Kirwan B.A., Anker S.D. (2020). Ferric carboxymaltose for iron deficiency at discharge after acute heart failure: a multicentre, double-blind, randomised, controlled trial. Lancet.

[2] Anker S.D., Comin Colet J., Filippatos G. (2009). Ferric carboxymaltose in patients with heart failure and iron deficiency. N Engl J Med.

[3] Chang H.C., Wu R., Shang M. (2016). Reduction in mitochondrial iron alleviates cardiac damage during injury. EMBO Mol Med.

[4] Kali A., Cokic I., Tang R. (2016). Persistent microvascular obstruction culminates in the confluence of iron oxide nanocrystal formation, proinflammatory burden and adverse left ventricular remodeling in chronic myocardial infarction. Circ Cardiovasc Imaging.

[5] Inserte J., Barrabés J.A., Aluja D. (2021). Implications of iron deficiency in STEMI patients and in a murine model of myocardial infarction. J Am Coll Cardiol Basic Trans Science.

